# Translating Research on Myoelectric Control into Clinics—Are the Performance Assessment Methods Adequate?

**DOI:** 10.3389/fnbot.2017.00007

**Published:** 2017-02-14

**Authors:** Ivan Vujaklija, Aidan D. Roche, Timothy Hasenoehrl, Agnes Sturma, Sebastian Amsuess, Dario Farina, Oskar C. Aszmann

**Affiliations:** ^1^Clinic for Trauma Surgery, Orthopaedic Surgery and Plastic Surgery, Research Department for Neurorehabilitation Systems, University Medical Centre GöttingenGoettingen, Germany; ^2^Department of Bioengineering, Imperial College LondonLondon, UK; ^3^Christian Doppler Laboratory for Restoration of Extremity Function, Medical University of Vienna, ViennaAustria; ^4^Department of Physical Medicine, Rehabilitation and Occupational Medicine, Medical University of Vienna, ViennaAustria; ^5^Master Degree Program “Health Assisting Engineering”, University of Applied Sciences FH Campus Wien, ViennaAustria; ^6^Otto Bock Healthcare Products GmbH, ViennaAustria; ^7^Division of Plastic and Reconstructive Surgery, Department of Surgery, Medical University of Vienna, ViennaAustria

**Keywords:** myoelectric prosthesis, prosthetic assessment, myoelectric control, SHAP, box and blocks

## Abstract

Missing an upper limb dramatically impairs daily-life activities. Efforts in overcoming the issues arising from this disability have been made in both academia and industry, although their clinical outcome is still limited. Translation of prosthetic research into clinics has been challenging because of the difficulties in meeting the necessary requirements of the market. In this perspective article, we suggest that one relevant factor determining the relatively small clinical impact of myocontrol algorithms for upper limb prostheses is the limit of commonly used laboratory performance metrics. The laboratory conditions, in which the majority of the solutions are being evaluated, fail to sufficiently replicate real-life challenges. We qualitatively support this argument with representative data from seven transradial amputees. Their ability to control a myoelectric prosthesis was tested by measuring the accuracy of offline EMG signal classification, as a typical laboratory performance metrics, as well as by clinical scores when performing standard tests of daily living. Despite all subjects reaching relatively high classification accuracy offline, their clinical scores varied greatly and were not strongly predicted by classification accuracy. We therefore support the suggestion to test myocontrol systems using clinical tests on amputees, fully fitted with sockets and prostheses highly resembling the systems they would use in daily living, as evaluation benchmark. Agreement on this level of testing for systems developed in research laboratories would facilitate clinically relevant progresses in this field.

## Introduction

Recent progresses in active prosthesis control for the upper limb include the introduction of novel control approaches (Scheme and Englehart, 2011; Jiang et al., 2014a; Amsuess et al., 2016), sensor types and sensor fusion algorithms (Weir et al., 2003; Dosen et al., 2010; Cipriani et al., 2014; Ortenzi et al., 2015; Nissler et al., 2016), surgical techniques (Kuiken et al., 2004; Aszmann et al., 2015), as well as advanced hardware (Cipriani et al., 2011; Grebenstein et al., 2011; Catalano et al., 2014). Nonetheless, the impact of these advances towards improving the experience of the everyday end user is still limited. The discrepancy between myoelectric solutions which academia develops and promotes, and the systems available on the market is indeed substantial. This issue has been previously discussed (e.g., Hill et al., 2009; Jiang et al., 2012; Farina and Aszmann, 2014) and relates to the conditions in which new methods are tested.

The necessity for testing prosthetic solutions in a greater number of amputees than currently done is a widely recognized problem. Moreover, the tests used often fail to include clinically relevant metrics. Performance metrics prevalent in laboratory research may be poorly associated to the clinical outcome, as noted previously (Simon et al., 2011; Jiang et al., 2014b; Ortiz-Catalan et al., 2015). In this perspective article, we support these arguments to further substantiate the relevance of this problem.

Transferring myoelectrical systems developed in the laboratory to clinical settings is a challenge that requires multidisciplinary efforts. Clinical tests, although not ideal, offer the most realistic prediction of the system performance in the daily use. These tests account for several of the challenges that laboratory-based assessment methodologies tend to neglect. For example, noiseless laboratory-based evaluation platforms fail to account for the end effector loads, poor socket fitting and sweating.

Here, we briefly introduce the evaluation methods regularly applied for prosthetics use, with a focus on offline approaches and some selected clinical measures. Moreover, we provide experimental data on seven conventional myoelectric users. The literature review and the experimental data are limited to the primary aim of providing our view on assessment procedures for myocontrol and suggestions for their improvement.

## Performance Evaluation

Laboratory-based techniques and tests for measuring the performance in controlling a myoelectric interface are numerous and, in case of offline techniques, have been mainly derived or adapted from the machine learning literature. On the other hand, initially, clinicians have mostly adapted established hand and arm impairment assessment tools to the evaluation of functional recovery with prostheses. However, in recent years, new clinical measures have been introduced to specifically target the amputee patient population.

### Laboratory Metrics

Evaluation and assessment techniques for myocontrol in strictly laboratory conditions can be broadly divided in two groups—those quantifying the system performance through offline metrics and those based on online assessments using virtual prostheses or games.

Depending on the type of the evaluated control algorithm, offline performance is most commonly assessed using either classification accuracy (Ortiz-Catalan et al., 2013) or the *R*^2^ error with respect to a given prompt (Ameri et al., 2014). The first approach relies on the number of correct estimates that the tested classifier makes, given the new, unseen data. The second compares the estimated command with respect to a reference cue. It has been shown that offline analysis fails to reflect the performance exhibited in online scenarios (Jiang et al., 2014b; Ortiz-Catalan et al., 2015). This is classically attributed to the fact that offline analyses do not account for adaptation of the user to non-stationary signal features.

Several virtual reality (VR) based assessment benches have been proposed in recent years. These systems simulate the online use of the prosthesis, at various levels of abstraction, while still being research-based settings. They offer the advantage of not dealing with the full implementation of the system, avoiding the challenges of socket design and hardware implementations. These VR systems are sometimes abstract with respect to the intended control (Ison et al., 2016) and commonly consist in steering a computer avatar in multiple directions to assess the performance when controlling specific degrees of freedom. Alternatively, computer games can be presented to the users, e.g., controlling a cursor to hit targets on a computer screen (Ameri et al., 2014; Jiang et al., 2014a). Finally, users can also be instructed to move a virtual arm into a target posture (Simon et al., 2011), as a part of an elaborate VR test bench.

The online systems are superior to the offline evaluations since they include the user in the loop and therefore account for his/her adaptation to the system. Parameters such as completion rate, path efficiency, number of overshoots or throughput, provide a solid quantitative evaluation of online performance. Further, the Fitts’ law (Fitts, 1954) has also been applied in evaluating myocontrol. It provides a single statistical measure to characterize online control (Fimbel et al., 2006; Park et al., 2008; Scheme and Englehart, 2013). Nonetheless, even if some of these test benches offer realistic testing scenarios, they have limitations. For example, weight bearing by the prosthesis and stump dynamics causing pressure changes within the socket fitting are important realistic factors of influence (Daly et al., 2014), not included in these tests. On the other hand, VR systems have found relevant applications in patient training (Roche et al., 2015; Sturma et al., 2015) and can be combined with table-top prosthetics (Stubblefield et al., 2011).

### Clinical Metrics

Clinical and rehabilitation specialists rely on a set of tests as well as questioners for assessing the user performance in myoelectric control. These tests prompt users to manipulate a variety of objects and to execute tasks mimicking those of daily living. The majority of the clinical scores validate the capability of executing certain tasks by quantifying the completion time. A battery of clinical tests requires the presence of certified examiners.

The box and blocks (B&B) test is one of the simplest and most commonly used clinical tests for evaluating the severity of upper limb deficiency. It consists of transporting, one by one, a number of square wooden blocks over a barrier using the prosthesis. The quantitative performance index for this test is the number of blocks that are successfully moved in a fixed time interval (usually 1 min). This test is simple to implement but only focuses on a limited number of DoFs and requires a minimal skill by the user.

The Clothes Pin Relocation Test (CPRT) requires the user to move a set of clothes pins of various resistances from a horizontal to a vertical bar. Since this is primarily a rehabilitation tool, the exact evaluation procedure has not been defined yet. However, most therapists use four clothespins of different resistances (1, 2, 4 and 8 lbs) and prompt the subjects to relocate them from the lowest horizontal bar to the most convenient position on the vertical bar. The time of execution is then recorded from the starting neutral position to the final neutral position. The CPRT requires activation of several degrees of freedom, although it often promotes compensatory movements which are not accounted for in the final outcome score.

The Southampton Hand Assessment Protocol (SHAP) is one of the most elaborate hand impairment evaluation tests (Light et al., 2002). It consists of 26 individual tasks that include six grips and their combinations. It can be separated into abstract object handling and execution of activities of daily living (ADL). Its final outcome is a number in the range 0–100, where 0 corresponds to absence of hand function and 100 to a healthy hand function, which mainly reflects the time needed for completing the tasks. SHAP is a very detailed hand assessment tool and therefore it tends to be lengthy and tiring for the patients, especially those with limited capabilities. Additionally, it mainly quantifies the time needed for execution and does not account for the way in which the tasks are completed.

The Action Research Arm Test (ARAT) is a global arm function assessment procedure. It is divided into four sub-scales—grasp, grip, pinch and gross movement—that evaluate abstract object manipulation strategies. The maximum ARAT score is 57, corresponding to normal upper limb function. This score is based on the opinion of certified examiners that rate the quality of execution of each task on a scale from 0 (cannot perform) to 3 (performs normally).

In addition to the above, several other clinical tests and questioners have been devised targeting different functions and ways of assessing upper limbs, such as the Assessment of Capacity for Myoelectric Control (ACMC; Hermansson et al., 2005) and the Jebsen-Taylor Test of Hand Function (JTHF; Davis Sears and Chung, 2010). Contrary to the other tests discussed, ACMC is a clinical evaluation test specifically tailored for myocontrol rather than generically for hand function. Nonetheless, it suffers of a relatively large subjective component which has so far limited its use.

Although being the best test bench available so far, existing clinical tests are still limited in fully representing the functional benefit of the prosthetic system for the patients. The main limitation that needs to be addressed in the field is the lack of objective clinical metrics to quantify the way movements are performed with respect to natural motor tasks. Different control algorithms may score similarly for clinical tests that quantify the time needed to perform a set of standard tasks but yet provide very different ability for the user to perform movements with natural postures (Aszmann et al., 2016).

## Experiments

We provide data on amputees that compare the accuracy estimated offline, for one of the classic control schemes developed over the past decades, with clinical scores. These data serve the purpose of representatively supporting the need for clinical tests for myocontrol developments. Therefore, the experiment and results do not aim at providing general conclusions on all myocontrol schemes and evaluation methods but rather at exemplifying the view presented in this perspective article.

Seven male transradial myoelectric users agreed to participate. They were all fit with custom-made sockets and with the Michelangelo hand (Ottobock Healthcare GmbH, Austria) with additional wrist rotation and flexion/extension units. The study was performed in accordance with the recommendations of the local ethics board of the Medical University of Vienna (Ethics Commission number 1044/2015), with written informed consent from all subjects. Subjects were fully briefed on the study protocol and possible adverse effects in presence of a clinical staff. All given consents are in accordance with the Declaration of Helsinki. All involved participants were transradial amputees with previous experience in using commercially available prosthetic devices. Before participation in the experiment medical state of each participant has been checked by the clinical staff.

The control of the prosthesis was based on the common spatial pattern (CSP) based classifier, as described by Amsuess et al. (2016). The EMG signals were recorded with 8 bipolar surface electrodes (Otto Bock raw signal electrodes 13E200 = 50AC). The control system allowed the subjects to access seven prosthetic functions—wrist flexion/extension, wrist pronation/supination, hand open, pinch, and key grip. All the motions were recorded in three arm positions (relaxed, fully extend arm in front of the ipsilateral shoulder, and fully extended arm across the contralateral shoulder) and at three forces (30%, 60% and 90% relative to the EMG level at maximum voluntary contraction force) while wearing the full prosthetic fitting. For offline accuracy assessment, the classifier was trained by data collected in only one arm position and tested against the remaining two data sub-sets. The average of the three scores was the reference performance of the subject. The entire data set was used for training the same CSP classifier that allowed execution of the B&B and SHAP tests. These particular clinical tests were chosen since they cover a wide range of assessment goals while being entirely objective. Additionally, these two tests have been widely recognized and familiar to academic and industry-based developers as well as clinical experts.

**Figure 1:**
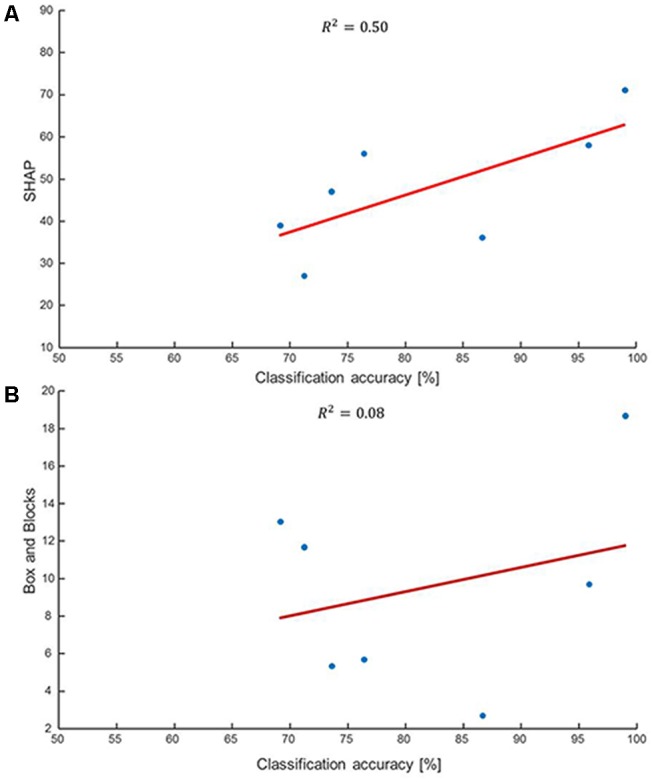
**Correlation between clinical scores and classification accuracies**. **(A)** Correlation between the clinical Southampton Hand Assessment Protocol (SHAP) score and offline classification accuracy. The offline scores have been obtained in realistic conditions with the patients wearing their prostheses and training and testing performed on sets of data obtained in different arm positions. Despite the realistic conditions, the associations shown here are not strong. For example, a SHAP score of approximately 40 may correspond to classification accuracy lower than 70% or greater than 85% depending on the user. The SHAP requires precise manipulation over short periods of time which is not captured by this offline metrics. **(B)** The correlation between the clinical Box&Blocks (B&B) test and the offline classification accuracy shows almost complete absence of association between the two. For instance, the two patients who achieved classification accuracies >95% were radically different for the number of blocks they could transfer. When computed in less realistic conditions (without prosthesis and testing on the same arm posture as training) the offline scores were greater than in the presented conditions but showed almost no correlation with clinical tests, since the majority of the patients were not able to conclude the clinical evaluation without substantial retraining.

**Figure 2:**
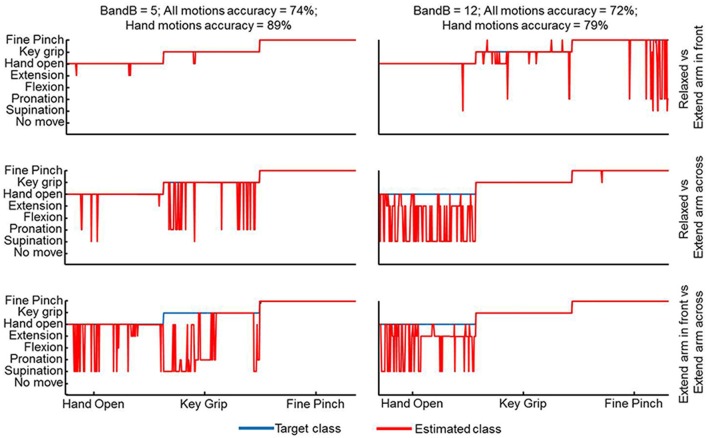
**Classification output for two patients with substantially different outcome of the B&B test but very similar classification accuracies over all motions.** The focus here is on the three hand motions that are most relevant for the B&B task—hand open, key grip and fine pinch. The offline accuracy for these motions is lower for the subject with the higher clinical score.

The performance scores in both offline and clinical tests are presented in Figure 1. The offline classification accuracies are slightly lower than in other studies (Ahsan et al., 2010; Liu et al., 2013) because of the different arm positions used for training and testing as well as the full prosthetic fitting which is not usual in offline evaluation studies. Although with these choices we have presumably maximized the prediction capacity of offline indexes for clinical scores, still the clinical scores did not strongly correlate with the offline performance measures. For example, there were two patients who achieved a similar SHAP score just below 40 but with very different classification accuracies of <70% and >85% (Figure 1A). Similarly, two patients who had similar classification accuracies of 70%–75% had SHAP scores of 27 and 47 (Figure 1A). The B&B test requires less skill to be performed than the SHAP. However, the B&B score was even less associated to the offline classification than the SHAP (Figure 1B). For example, subjects with an offline accuracy >95% performed very differently in this test (Figure 1B). Furthermore, when considering strictly the hand movements—hand open, fine pinch and key grip—that are primarily used for this test, the mismatch between this test and offline performance was even more substantial. This was observed consistently in all patients but it is shown representatively for only two patients in Figure 2. For these patients, the average classification rate across the three hand motions was 89% and 79% whereas the transferred blocks (score of the B&B) were 5 and 12, respectively.

When the offline evaluation was performed by using data collected without wearing the prosthesis and tested on the same arm position as the training, as more commonly done in laboratory tests (e.g., Englehart et al., 1999; Hargrove et al., 2009; Li et al., 2010; Ortiz-Catalan et al., 2014b), the resulting offline classification rates were high and comparable to those reported in the literature (>90% on average). However, once fully fitted, the majority of patients were unable to successfully conclude the clinical evaluations without retraining, suggesting that the classic offline evaluation procedure performed in several research studies, even though indicative, does not necessarily vouch for superior clinical performance.

## Discussion

Abandonment rates among upper limb myoelectric prosthetic users are still very high (Burrough and Brook, 1985; Glynn et al., 1986; Østlie et al., 2012). At the same time, research efforts have provided several new solutions for myocontrol that have been proven to be highly functional strictly under laboratory conditions. The limited transfer from research to real world applications likely depends on an insufficient level of evaluation procedures.

Using novel prototypes of myoelectric systems in daily life would provide the ultimate assessment, but this strategy would often require official certification by notified bodies, which often goes beyond the possibilities of academic development. The COAPT system (Coapt LLC, 2016) is one of the first systems that has reached this level of testing. Clinical evaluations at earlier stages are a compromise between laboratory conditions and real-life tests. Although not perfect, clinical tests are closer to the conditions of interest for the users than offline assessments or online tests using virtual prostheses which provide valuable, but not always sufficiently transferable scores. Here, we have presented an example of this dissociation on a small sample of amputees and focusing on offline metrics, for demonstration purposes. We have compared clinical scores with offline indexes of performance extracted in the most realistic offline conditions (patients wearing a prosthesis, training and test sets obtained on different arm postures). Despite these conditions rarely being met in the offline studies, the prediction capacity for clinical outcome was not strong. On the other hand, when the offline indexes were obtained in more common laboratory conditions without the prosthesis and for the same arm posture for test and training, the clinical information they provided was minimal (indeed with this training, once fitted with the prosthesis patients could not even finish the clinical tests without re-training). Further extrapolating, it is obvious that an offline analysis performed in these simple conditions and, in addition, on able-bodied individuals instead of patients, is of rather poor clinical value. While we are fully aware that in the initial evaluation of a new myocontrol scheme the strict laboratory tests on healthy individuals are valuable and needed for assessing the basic algorithmic working principles, there is also the need to make efforts in continuing the evaluations of promising algorithms in clinically-relevant settings (and to further develop clinical tests that fully represents the functional benefits). We believe that the evaluation stages after the laboratory level have had so far a slower progress, and less academic interest, with respect to the proposal of new algorithms.

Considering the discrepancy presented in the literature (Jiang et al., 2014b; Ortiz-Catalan et al., 2015) and further supported here, it seems necessary that novel myoelectric systems that passed laboratory testing are then fully clinically evaluated for assessing their performance. For this purpose, researchers and clinicians should jointly devise a standardized testing framework for quantitatively and qualitatively assessing the performance of upper limb prosthetic devices and their users to boost the process of commercialization and, as a consequence, availability for the patients. This need does not only relate to the feed-forward control aspects, on which we focused here, but also to fully closed-loop systems that include sensory feedback integration (Gonzalez and Yu, 2009; Jorgovanovic et al., 2014; Ortiz-Catalan et al., 2014a).

## Author Contributions

IV, DF and OCA: substantial contributions to the conception; IV, ADR, SA, DF and OCA: design of the work; IV, ADR, TH, AS and SA: the acquisition; IV, ADR, TH, AS, SA, DF and OCA: analysis; IV, ADR, TH, DF and OCA: interpretation of data for the work; IV, ADR, DF, OCA: drafting the work and revising it critically for important intellectual content . The major writing of the report was completed by IV, DF and OCA. Final approval of the version to be published was given by all authors.

## Funding

This work was supported by the European Union’s Horizon 2020 research and innovation program under grant agreement number 687795 (project INPUT) and by the Christian Doppler Research Foundation of the Austrian Federal Ministry of Science, Research and Economy.

## Conflict of Interest Statement

The handling Editor declared a past collaboration with one of the authors DF and states that the process nevertheless met the standards of a fair and objective review. The other authors declare that the research was conducted in the absence of any commercial or financial relationships that could be construed as a potential conflict of interest.
